# Supplementary material for: An updated floristic map of the world
Source: Nat Commun. 2023 May 30;14:2990. doi: 10.1038/s41467-023-38375-y (PMC10229591; doi:10.1038/s41467-023-38375-y)
Supplement: Supplementary file 1 — Supplementary Information [file 41467_2023_38375_MOESM1_ESM.pdf]

SUPPLEMENTARY METHODS

The phylogeny of global angiosperm genera

We downloaded all the sequence data of seed plants available in GenBank (as of May 19, 2018) for the following genes commonly used in plant phylogenetic studies: 18S ribosomal DNA (18S rDNA), internal transcribed spacer region (ITS, including ITS1, 5.8S ribosomal DNA and ITS2), and 26S ribosomal DNA (26S rDNA) from the nuclear genome; ATPase β-subunit gene (atpB), Maturase K (matK), NADH dehydrogenase F (ndhF) and ribulose bisphosphate carboxylase large chain (rbcL) from the chloroplast genome; and Maturase R (matR) from the mitochondrial genome. This gene sample represents both fast (e.g., ITS) and slowly (e.g., 18S rDNA, rbcL, matR) evolving genes. When multiple sequences were available for a genetic marker of a species, the longest one with the latest publication time was kept. Sequences from hybrids, unidentified taxa and taxa with dubious identification were not included. Accession number of the sequences that were used in our molecular analyses are available in Supplementary Data 6.

To construct the genus level dataset, we firstly assessed the monophyly of each genus with sequence data using the large species-level phylogeny generated by [1]. For the monophyletic genera, one representative sequence per genetic marker per genus was used as the placeholder of the genus. A total of 593 genera (4.7%) were identified as non-monophyletic. 1) For the non-monophyletic genera caused by very few stochastic intruders from other genera, we removed the intruders' sequences from our database. 2) For non-monophyletic genera with several clades, we identified all the monophyletic clades and estimated the number of species included in the tree of [1] for each clade. Then the largest clade of each non-monophyletic genus, which represented the majority of the genus, was used to represent the genus. 3) For polyphyletic genera, we only selected species from the core monophyletic clades of each genus. These procedures ensured that we only combined sequences from species that belonged to the same monophyletic lineage. The final matrix included molecular data for 12,539 seed plant genera.

Because the alignment of some gene regions (particularly the ITS1 and ITS2) between very divergent groups is difficult and may lead to unwanted artefacts, we adopted an alignment strategy with the following steps. 1) The sequences of each plant order were placed in a separate matrix and were aligned separately using L-INS-i

strategy in MAFFT v7.4 with the following parameters: --localpair -- maxiterate 1000 –adjustdirectionaccurately. 2) The order-level alignments were put together as a single fasta file. For each of these combined fasta files,subMSAtable file with the information on which sequences correspond to individual order-level alignments was created using the makemergetable.rb script distributed with MAFFT v7.4. 3) The order-level alignments were then merged and refined cross orders using using the --localpair --merge commands in MAFFT v7.4. This approach decreases alignment errors and ensures higher consistency with currently accepted taxonomy (i.e. APG IV).

For the further phylogenetic analysis, to ensure better consistency with the higher-level seed plant relationships in APG IV, we constrained the phylogenetic analyses in RAxML v8.0.26 using the APG IV relationships among angiosperm orders and among eudicots, monocots and magnoliids. Similarly, recent comparative studies of angiosperms have used family-level topology to constraint for their phylogeny reconstruction[2]. Here we apply the constraints on the topology at the deeper nodes of the tree (i.e., at order level), as these are more likely to present problems given the character sample for this study (for example, some of these relationships have been elucidated using morphological data that is not used in our analyses).

Phylogenetic analysis were partitioned by RAxML v8.0.26[3] and the GTRGAMMA model. The tree was dated with the program treePL v1.0 [4]. The fossil calibration points used here followed [2]. Because the crown age of Angiosperms is still debated [5], and different studies have provided estimates that vary from a maximum of about 280 Ma [6] to a minimum of 130 Ma [7], we conducted three different dating analyses with different constraints on the age of the Angiosperm crown as the following: 1) min = 149 Ma, max = 256 Ma based on [8]; 2) min = 140 Ma, max = 210 Ma based on [9]; and 3) min = 140, max = 150 Ma as suggested by [10].

We checked whether families as defined in the APG IV were recovered monophyletic by our molecular phylogeny reconstructed. In the 423 families included in our dataset with molecular data, 404 (95.5%) families were recovered as monophyletic in our phylogeny. Only 19 (4.5%) families were found to be nonmonophyletic and most of these cases were due to the position of a few (often a single) genera, while core species of these families reman monophyletic (see Supplementary Data 4 for the list of non-monophyletic families). Families that were not recovered monophyletic are also families in which generic composition has been

contentious (e.g. Capparaceae[11]). Therefore, our phylogeny offers a good global overview of the Angiosperm phylogeny at the genus level.

To explore the potential influence of the fast-evolving genes (particularly ITS1 and ITS2) on phylogenetic topology, we reconstructed the phylogeny using sequence data without ITS conducted in RAxML v8.0.26 and dated it in the same method as described in the previous paragraph. Then we compared it with the phylogeny based on the full sequence dataset. We found that only 9.4% genera (1,176 genera of the total 12,456 of angiosperm genera with molecular data) were dropped when removing ITS from the reconstruction (see Supplementary Fig. 18a for the proportion of dropped genera in each family). The dropped genera were mainly from a few very large families (Supplementary Fig. 18b, e.g., Asteraceae, for which 267 out of 1454 genera were dropped), while the number of genera in 349 (82.5%) out of 423 families remained unchanged after removing ITS. Most importantly, the removal of ITS did not affect the overall topology of phylogeny. Specifically, we found that the topologies of 404 families (95.5% of all 423 families) remained the same after removing ITS. Some small changes in the topology of a few genera were found in the very large families. Moreover, we found that after removing ITS, the branch lengths of genera and the phylogenetic distances between genera, which is directly related with the estimation on phylogenetic beta diversity, were highly consistent with those estimated from the original phylogeny (Supplementary Fig. 18c, d; for branch lengths of genera, Pearson $r = 0.81$; for phylogenetic distances between genera, Mantel $r = 0.965$, $p = 0.01$). These results suggest that the removal of ITS did not dynamically change the phylogeny and the estimation of phylogenetic beta diversity used for the division of biogeographic regions in the following analysis.

After the molecular was dated, other currently described seed plant genera without sequence data were added to the dated phylogeny as polytomies based on current taxonomy, and then were resolved using the polytomy resolver following [12] conducted with BEAST v1.8.0 [13]. For the BEAST analyses we used a birth-death model with uniform priors for the mean growth rate ($\lambda - \mu$) and the relative death rate (μ/λ) parameters. The model was run for 11 million generations and the posteriors were sampled every 1000 generations. After discarding the first 10% of generations as burn-in, we used Tracer v1.6.0 [14] to examine the effective sampling sizes (ESS) of all parameters, chain mixing and convergence to a stationary distribution. The post burn-

in sample showed convergence to a stationary distribution, good mixing and high ESS for all parameters (ESS > 200). Following [15], the final ultrametric topologies that included all described angiosperm genera were produced based on the maximum clade credibility tree extracted from the post-burn-in sample.

Here we extracted the angiosperms from this seed plant phylogeny for further analysis, which contains 14,244 genera in total. We repeated the clustering and ordination analysis using the three phylogenies with different constraints for the crown age of angiosperms, and the results were highly consistent (see Supplementary Fig. 3). In the main text we presented the results based on the phylogeny with a constraint of 140-210 Ma for the crown age of angiosperms as recent family-level phylogenies of angiosperms suggest that the crown age of angiosperms is ca. 190-210 Ma [16,17] and those based on phylogenies with alternative dating schemes as supplementary materials. It is noteworthy that all of the results based on the phylogenies with different scenarios of angiosperm crown age are highly consistent.

The phylogenies used in our study represent the most recent and most complete angiosperms genus-level phylogeny. Currently there are three published large angiosperms phylogenies [1,2,18]. However, the coverage of angiosperm genera in these phylogenies (i.e. 8180 [2], 10,155 [1], and 8399 genera [18]) is significantly lower than that of the phylogenies used in this paper which include molecular data for 12,539 genera. The divergence time estimation of these phylogenies also differs. Although these phylogenies are based on a largely overlapping set of fossil calibrations and the same dating algorithm [1,2,18], all except the phylogeny used here are based on a single dating scheme which does not allow to test the potential effect of alternative hypotheses for the crown age of angiosperms on our findings. In contrast, the phylogenies used here have been dated with three different constraints for the crown age of angiosperms. Finally, although unsequenced taxa are included in both [1] and the phylogenies used here based on current taxonomy, these unsequenced taxa have been kept as unresolved polytomies in [1], which makes it difficult to test the potential influences of the relatedness between these unresolved taxa on our findings.

Global distributions of angiosperm genera

The geographic standard units (hereafter GSUs) used for the compilation of species distribution data were generated following the Global Administrative Areas

(GADM) database version 1 (https://gadm.org/data.html) and the World Geographic Scheme for Recording Plant Distributions (WGSRPD) as the base maps [19]. To reduce the effects of data deficiency and area on the estimation of genus richness, we merged small adjacent regions of WGSRPD and GADM into larger ones and splitted the large units of WGSRPD to small ones based on GADM. The final size of each GSU is ca. 4° longitude × 4° latitude (366,179.4 ± 20,245.85 km^2). Small islands (<25,000 km^2) and the Antarctica were removed. Finally, the earth's landmass was divided into 420 GSUs (see Supplementary Data 2 for the detailed information of GSU). This scheme of geographic standard units has been used for the compilation of species distribution data in several previous studies [19,20]

Distributional records for angiosperms were compiled at the species level from over 1,100 data sources, including published regional and local floras, floristic investigations, specimen records and online databases (see Supplementary Data 1 for the full list of data sources). We classified the raw distributional data into four types: coordinates, range maps, gridded distributions, and recorded localities. Depending on the types of the raw data, we applied different methods to reduce spatial conflicts between the original records and the boundaries of the GSUs used in our study and to improve the accuracy of species distributions in the final dataset. For species distributions recorded as coordinates (e.g. GBIF), we mapped the coordinates to GSUs using the 'inpolygon' function in MATLAB v2017a. For species distributions recorded as range maps, we digitized and georeferenced these boundaries in ArcGIS 10.0 and then intersected them with our GSUs. For species distributions recorded as grid cells, we overlapped these grid cells with GSUs used in our study. The record of a grid cell was kept only when the intersected area of the grid cell was larger than half of its size. For the distribution data recorded as locality names, we searched them in the global geographic names service (http://www.geonames.org/) and then intersected the recorded boundary with the boundaries of GSUs in our study. When a recorded geographic unit intersected with more than one GSU, we assigned the record to the GSU that covered over 80% area of the recorded unit.

To improve the quality of species distribution data, we set a threshold for the number of data sources to keep an occurrence record of a species in a given GSU. For geographical units in Europe, Australia, China, South Africa, Madagascar and North America, an occurrence record of species in a geographical unit corroborated by at least

3 data sources was retained, leading to high confidence of the data quality in these regions. For the geographical units in Central America, Greenland, Amazon and Turkey, an occurrence record of species in a geographical unit corroborated by at least 2 data sources was retained, leading to medium confidence of the data quality in these regions. The entire data was retained for India, North and Central Africa, and Patagonia because of data deficiency in these regions, leading to relatively low confidence of the data quality in these regions.

The taxonomic status and the accepted names of species from all data sources were standardized following the World Flora Online (WFO, http://www.worldfloraonline.org/, accessed: December, 2022), Catalogue of Life (COL, https://www.catalogueoflife.org/, accessed: May, 2018), ThePlantList (TPL) available at http://www.theplantlist.org/ (accessed: Jan 3, 2015) and POWO (accessed: December, 2022). We kept accepted names with the highest confidence level. Taxonomic names that were identified as 'unresolved' in both COL and POWO were removed. Synonyms and misspelt names are replaced with the corrected/accepted names. The misspelt taxonomic names were corrected using the Taxonomic Name Resolution Service 4.0 (TNRS, https://tnrs.biendata.org/), which has been widely used in plant studies.

During data compilation, we also collected the status of species (i.e. being native, cultivated, introduced, invasive and hybrid) from regional data sources as much as we could, and non-native and hybrid species in different regions with clear records were not included in the database. After the compilation of distribution data at species level, we further checked the distribution maps and removed cultivated records from the database following the Plants of the World Online (POWO, https://powo.science.kew.org/, accessed: May, 2019) and efloras (http://www.efloras.org/, accessed: May, 2019).

Finally, we compiled the distributional data for each genus by aggregating distribution data of all its species. The distribution maps of all genera were carefully verified manually by the members in the institutes of the last authors of this paper to improve the quality of the distribution data. We then integrated the phylogeny and distribution data and the final distributional database contains 384,771 records for 12,664 angiosperm genera (see Supplementary Data 2), representing 90.63% of the total 13,974 accepted genera in POWO [21].

Taxonomic and phylogenetic beta diversity

Simpson beta diversity [22] modified by [23] was used to represent the dissimilarity in species assemblages among GSUs (Equation 1).

$$\text{Beta diversity} = 1 - \frac{a}{a + \min(b,c)} \qquad \text{Equation (1)}$$

where *a* represents species shared between two geographic standard units, *b* and *c* represent species unique to each unit. We used this metric because it is not affected by the hierarchical data structure and provides unbiased estimation of compositional turnover across space [24,25]. Pairwise matrices of taxonomic beta diversity (calculated using the number of shared (a) and unique species (b, c) between two geographic standard units) and phylogenetic beta diversity (calculated based on the length of shared (a) and unique branches (b, c) of the overall phylogeny in each pair of geographic standard units) were generated for further clustering analysis using the above equation.

Floristic domains and sub-domains identified by hierarchical clustering

Hierarchical clustering analyses were conducted to group GSUs into floristic realms and sub-realms. With this method, the GSUs with the highest similarity (i.e. lowest distance) were first grouped together and then the most similar groups were grouped into clusters. This process was repeated until all GSUs were all grouped into a single cluster. Then a dendrogram was generated by the clustering analysis, which represented the hierarchical relationships among floristic realms and sub-realms. The hierarchical clustering analyses were conducted using the "hclust" function in stats (version 3.6.2) package in R 3.6.1 [26] and for the matrices of taxonomic and phylogenetic beta diversity between the GSUs, separately. The whole procedure can be divided into two main parts as follows.

Part 1 Choosing a clustering algorithm

In the hierarchical analysis, several methods are widely used to estimate the distances between clusters. Thus, we need to choose the best algorithm prior to do the formal clustering analyses. Here, we compared seven clustering algorithms provided by the "hclust" function, "single", "complete", "median", "mcquitty", "average", "centroid" and "ward.D2", respectively. We chose the best one by comparing the performance and accuracy of these seven methods using the following approach

proposed by Holt et al. [25].

(i) Performance evaluation. This evaluation aims to choose the clustering algorithm that can best represent the floristic divergences with the lowest number of clusters. First, using each of the seven clustering algorithms, we grouped the GSUs into different numbers of clusters and calculated the between-cluster beta diversity. Second, we calculated the proportion of beta diversity explained by identified clusters (P_{beta}) as the sum of between-cluster beta diversity divided by the sum of the whole beta diversity matrix following [25]. Third, we demonstrated the variations in P_{beta} with the increase in the number of clusters for each algorithm. In general, with the increase in the number of clusters, the P_{beta} increases. Following [25], the best performing algorithm was identified as the one returning the minimum number of clusters when the P_{beta} reached 99%.

(ii) Accuracy evaluation: This evaluation aims to choose the clustering algorithm that can represent the floristic divergences with the lowest biases. To do this, the co-phenetic correlation coefficients were calculated for each clustering algorithm using the "cophenetic" function in stats (version 3.6.2) package [27] in R 3.6.1 [26]. We firstly obtained the co-phenetic distance matrix by calculating the branch length distances separating each pair of tips (i.e. GSUs) on the dendrogram tree. Then the co-phenetic correlation coefficients were estimated as the Pearson product-moment correlation coefficient between the co-phenetic distance matrix and the beta diversity matrix between all GSUs. The clustering algorithm with the highest accuracy has the highest co-phenetic coefficients.

The above evaluations indicated that the "average" algorithm outperformed all tested alternatives (Supplementary Fig. 14). Therefore, the "average" method, also known as the Unweighted Pair Group Means Algorithm (UPGMA), was finally chosen for defining floristic realms and sub-realms of the global angiosperms.

Part 2 Identifying floristic realms and sub-realms

All the GSUs were clustered into different clusters using UPGMA. The clustering process and the hierarchical relationships among clusters were shown using the UPGMA dendrogram. We cut the UPGMA dendrogram to get the minimum number of clusters required to reach P_{beta} = 80%. These clusters of GSUs were then defined as floristic realms. Similarly, the UPGMA dendrogram was cut to get the minimum

number of clusters required to reach P_{beta} = 95%, and these clusters were then defined as floristic sub-realms. Finally, we identified eight floristic realms and 16 floristic sub-realms. To illustrate the relationships between different floristic domains in a two-dimension non-hierarchical space, we used the Nonmetric Multidimensional Scaling (NMDS hereafter) with Kruskal's neighbor-joining algorithm based on the beta diversity matrices among all GSUs [28]. In NMDS plots, GSUs belonging to the same floristic realm were shown in the same color, and more similar GSUs occurred closer to one another.

The geographic sizes of the identified floristic realms and sub-realms are similar to those of the "Kingdoms" and "Regions" in Takhtajian's floristic map [29] (see Supplementary Table 1 for the detailed comparison). It should be noted that the methods used here for defining floristic clusters are different from Takhtajian [29]. Takhtajian's floristic "Kingdoms" and "Regions" are defined by endemic taxa that represent the distinctiveness of different floras [29]. Specifically, floristic "Kingdoms" are usually characterized by endemic families, subfamilies, and tribes; and floristic "Regions" are characterized by endemic genera (in few cases by endemic families). Thus, we used different terminology i.e., floristic realms and sub-realms.

Sensitivity analyses for the uncertainty in identifying floristic realms

The GSUs on boundaries between floristic realms may contain mixed floristic components, which may lead to less "hard" boundaries [30,31]. Meanwhile, floristic regionalization may also be influenced by 1) phylogenetic uncertainty and 2) incomplete sampling across lineages [32]. Thus, we conducted the following analysis to evaluate the uncertainties.

(i) Regions with uncertainty in floristic realm division. Following [25], we conducted the silhouette analysis to evaluate the performance of clustering. Specifically, the silhouette widths for each GSU was calculated as following:

$$s_i = \frac{b_i - a_i}{max(a_i,b_i)} \quad \text{Equation (2)}$$

where s_i is the silhouette width of a GSU *i*; a_i is the average dissimilarity between *i* and all other GSUs of the identified floristic realm to which *i* belongs; b_i is the average dissimilarity between *i* and all GSUs of the nearest realm to which *i* does not belong. In general, higher positive silhouette widths indicate better classification of a GSU into a floristic realm [33]. In contrast, GSUs with negative silhouette widths are

identified as uncertain regions that may contain mixed floristic elements [33]. As a result, most GSUs have positive silhouette widths, indicating that they are assigned to a floristic realm appropriately (Supplementary Fig. 2). Only a few regions near the boundaries between the Saharo-Arabian and Holarctic realms, between the Indian-Malaysian and Holarctic realms, and between the Neotropical and Chile-Patagonian realms have negative silhouette widths.

Thirdly, we assessed the robustness of our results to the choice of analytical approach by redefining the floristic realms using fuzzy c-means clustering[34]. Unlike the above-mentioned UPGMA method which assigns GSUs exclusively to one cluster, fuzzy c-means method estimates the likelihood of each GSU belonging to a certain cluster, under a given degree of fuzziness[35]. We conducted fuzzy c-means using "funny" function in the "cluster" package (version 2.1.4) [36] in R 3.6.1[26]. The number of clusters was set to 8 to match the number of realms in the UPGMA analysis. We repeated silhouette analysis to evaluate the clustering performance (see Supplementary Results and Discussion for the detailed comparison with UPGMA clustering results).

(ii) Phylogenetic uncertainty. As mentioned above, we repeated the clustering and ordination analysis using the tree dated with three different constraints for the crown age of angiosperms. Furthermore, to evaluate the potential impact of phylogenetic uncertainty in the placement of genera which positions were resolved using polytomy resolver [12] and BEAST v1.8.0, we repeated our regionalization analyses using: 1) the single maximum credibility tree, 2) the phylogenetic trees only containing taxa with molecular data, and 3) five randomly sampled post burn-in posterior trees from the polytomy resolver. The results based on the phylogenies with different scenarios of angiosperm crown age, the molecular phylogenies and post burn-in posterior phylogenies are all highly consistent (see Supplementary Fig. 3-8).

(iii) Incomplete sampling. A recent species-level floristic regionalization conducted by Carta et al. (2022) was based on a fraction of vascular plants [32]. In order to evaluate the effect of incomplete sampling, we extracted the angiosperm genera included in Carta et al. (2022) (9,905 angiosperm genera) and then we conducted the same genus-level regionalization as reported in our study. We found that incomplete sampling did not change our floristic maps (Figure S9, also see Supplementary Results and Discussion).

Changes in floristic realms through time

To explore the phylogenetic depth at which the spatial divergence of floristic assemblages appears, we conducted the following steps. First, we cut the phylogenetic trees at different geological times, including late Jurassic (160 Ma), Cretaceous (140 Ma, 120 Ma, 100 Ma and 80 Ma), Paleogene (60 Ma, 50 Ma, 40 Ma and 30 Ma), Neogene and Quaternary Periods (20 Ma, 10 Ma, 15 Ma, 10 Ma, 5 Ma and 0 Ma), and then collapsed all the descendent leaves of each branch encountered at each time, respectively. Second, the current geographic extension of an ancestral branch at a given time is represented by merging the geographic distributions of all its descendent leaves. Third, we calculated the phylogenetic beta diversity between different GSUs at different geological times following the methods of [37,38]. Fourth, we applied the UPGMA clustering method to generate floristic realms at each geological time. The floristic realms were identified by cutting the UPGMA dendrograms to return the minimum number of clusters require to get P_{beta} = 80%. Lastly, we used the NMDS ordinations to investigate the temporal changes in the dissimilarity of phylogenetic compositions between different realms following the method of [38,39]. Specifically, we illustrated the changes in the dissimilarity of floristic realms by overlaying the coordinates of the NMDS ordinations conducted for different geological times. This approach exploring the phylogenetic depth of the emergence of floristic realms have also been applied in previous studies [37-41]. Note that we do not intend to estimate the ancestral geographic ranges of phylogenetic branches or the ancestral floristic assemblages.

Effects of contemporary climate on floristic realm divisions

If the contemporary climate dominates the division between the identified floristic realms, the explanatory power of contemporary climate differences on the beta diversity between realms should be higher than those on the beta diversity within realms. The climate difference of a given pair of geographic standard units was defined as the Euclidean distance between them in a two-dimensional climatic space:

$$\text{Climate diff}_{i,j} = \sqrt{\left(\text{MAT}_i - \text{MAT}_j\right)^2 + \left(\text{MAP}_i - \text{MAP}_j\right)^2} \qquad \text{Equation (3)}$$

where *i* and *j* represent two given geographic standard units, and MAT and MAP represent annual mean temperature and annual mean precipitation, respectively. The values of climate are standardized prior to analysis.

To evaluate whether contemporary climate dominates the division between the identified floristic realms, we compared the effect of contemporary climate differences on the beta diversity between and within realms using Ordinary Least Square (OLS) regressions with phylogenetic beta diversity as the response variable and the corresponding contemporary climate differences as the predictor. Specifically, for each sister pair of floristic realms (or cluster of realms) (i.e. two adjacent realms or cluster of realms in the UPGMA clustering dendrogram), we built the following three OLS regression models:

$$\mathrm{PBD}_{\mathrm{im,jm}} \sim \mathrm{Climate\ diff}_{\mathrm{im,jm}} \qquad \text{Equation (4)}$$

$$\mathrm{PBD}_{\mathrm{in,jn}} \sim \mathrm{Climate\ diff}_{\mathrm{in,jn}} \qquad \text{Equation (5)}$$

$$\mathrm{PBD}_{\mathrm{im,jn}} \sim \mathrm{Climate\ diff}_{\mathrm{im,jn}} \qquad \text{Equation (6)}$$

where m and n respectively represent the two clusters of realms, im represents the ith geographic standard unit located in the realm (or cluster of realms) m, jn represents the jth geographic standard unit located in the realm (or cluster of realms) n, and PBD represents the phylogenetic beta diversity between the geographic standard unit i and the geographic standard unit j.

Equations 4-6 evaluated the explanatory power of contemporary climate differences on the beta diversity within a single realm (or cluster of realms) m or n and those on the beta diversity between the two realms (or clusters of realms), respectively. The Novozealandic realm contains only two basic geo-units and thus, we could not calculate R^2 for it.

Effect of historical climate and geographic isolation on floristic realms division

Plate tectonic dynamics could lead to changes in both geographic distance and dispersal barriers (e.g., ocean and mountains) among areas. These changes could subsequently lead to geographic isolations among floras, causing regional differences in evolutionary histories and result in distinct phylogenetic assemblages of plants. By combining all the paleo-digital elevation models obtained from Scotese's paleoatlas [42], we produced detailed paleogeographic maps with a spatial resolution of 1 × 1 degree from 80 Ma up to the present in 1 Ma steps following [43]. The changes in the paleo-locations and mean elevation of each 1-degree grid cell through time were estimated by combining the information on the dynamics of geological events involving sea floor spreading, continental rifting, continental collisions together with other indicators of

paleotopography and bathymetry [42].

Then, at every geological time interval of 1 Ma, we calculated the geographic isolation between each two geographic standard units following [44]. Specifically, we first estimated the coordinates of the center of each geographic standard units and identified their central grid cells at the present and in the geological history using the open source software GPlates v2.1 (http://www.gplates.org; [45]). The geographic isolation between two geographic standard units at each geological time was then estimated as the geographic isolation between their central grid cells, which was calculated using the paleogeographic maps obtained from Scotese's paleoatlas [42] and the 'cosDistance' function [46] in the 'gdistance' R package (version 1.6) [47]. This function assigned different conductance to different grid cells and then identified the route with minimum total route cost between a given pair of grid cells. The conductance of a grid cell was 0 in the ocean and was inversely proportional to its elevation on land. The cost value of a grid cell was calculated as the reciprocal of the mean conductance of all of its adjacent cells, where 0 was not included. The neighboring cells of a focal grid cell included those in 16 directions, including the four orthogonal and four diagonal nearest neighbors (the resulting graph is called the 'king's graph', because it reflects all the legal movements of the king in chess), and the eight neighbors that could be connected by 'knight's moves' in chess. For comparison, we also calculated the conductance without considering differences in elevation for the land, and it returns similar patterns of geographic distances; and we also calculated the cost value using alternative concepts of neighbors provided in the 'cosDistance' function, including the four orthogonal neighboring cells of a focal grid (4 neighbors); the four orthogonal and four diagonal nearest neighbors (8 neighbors, i.e., the 'king's graph' as mentioned above). And all alternative calculations yield similar patterns of distances (Person $r > 0.83$).

Climate can influence species distributions and phylogenetic compositions of a region through filtering effects and its effects on evolutionary history. Hence larger climatic differences between regions may lead to higher turnover in species composition and hence higher beta diversity. Paleoclimate from 150 Ma up to the present was reconstructed by [43] from the geographic distribution of lithological indicators of climate at a temporal resolution of 1 Ma and a spatial resolution of 1 × 1 degree. These indicators included coal, evaporite, bauxite, tillite, glendonite dripstones and the fossil records of important taxa such as high latitude occurrences of palms,

mangroves, and alligators [48,49]. The climate distance of a given pair of geographic standard units at a certain time interval was defined as the Euclidean distance between them in a two-dimensional climatic space (Equation 3). The MAT and MAP of a geographic standard unit at each geological time were estimated as the average value of all 1 × 1 degree grid cells within it.

To compare the relative effects of geographic isolation and climate distances between geographic standard units on the division of floristic realms, we calculated the Pearson correlation of geographic isolation and climate differences for each pair of floristic realms from 80 Ma to the present, and then conducted the hierarchical partitioning analysis using the following multiple regression model (Equation 7) with Gaussian residuals at each geological time separately:

$$\ln(\mathrm{PBD}_{\mathrm{im,jn}}) \sim \ln(\mathrm{Climate\ diff}_{\mathrm{im,jn,t}}) + \ln(\mathrm{Geographic\ iso}_{\mathrm{im,jn,t}}) \qquad \text{Equation (7)}$$

where *m* and *n* respectively represent a sister pair of floristic realms (or cluster of realms) , *im* represents the *i*th geographic standard unit located in realm (or cluster of realms) *m*, *jn* represents the *j*th geographic standard unit located in realm (or cluster of realms) *n*, t represents the given geological time, and PBD represents the phylogenetic beta diversity between the geographic standard unit *i* of realm (or cluster of realms) *m* and the geographic standard unit *j* of realm (or cluster of realms) *n*. Then the relative R^2 of geographic isolation and climate distances were estimated at each geological time, and its temporal trend was plotted to demonstrate the changes in the drivers of the divisions between floristic realms. Both geographic isolation and climate distances were standardized prior to conducting regression models.

Contribution of different clades on floristic realms division

The divisions between floristic realms are shaped by clades whose descendant lineages show little overlap in their geographic distributions. The contribution of a clade to the division between two realms was evaluated using node-based analysis developed by [41]. The node-based analysis calculates a 'geographic node divergence' (GND) score for each node in a phylogeny, which measures the degree of mismatch in the geographic distribution of the two sister lineages diverging at a given node, i.e., descendants of one sister lineage did not persist in the geographic range of the other [41]. Values of GND over 0.65 indicate significant distributional divergence between the two sister lineages, thus pinpointing the exact evolutionary splits associated with the division between

floristic realms. The GND score of a given node is calculated as a summary of multiple specific overrepresentation scores (SOS), one for each geographic unit occupied by the clade containing two descendant lineages. A SOS score is the z score of the relative overrepresentation by one of the two descendant lineages in the geographic unit, as compared to the expectation generated by a matrix-swapping null model. Values of SOS close to zero thus indicate that tips from both descendant lineages are equally represented in a given geographic unit, and positive (or negative) SOS values indicate predominance of one of the two descendant lineages (see Supplementary Fig. 15 for an example of the result of node-based analysis).

We extracted subtrees from the global angiosperm phylogenies for realm divisions. Each subtree contains all genera that are distributed in a given pair of realms. Then we used node-based analysis to detect the clades with GND scores over 0.65 and extracted the SOS scores of all the geographic standard units occupied by these clades. The contribution of each of these clades on the division of the two floristic realms was measured by the R^2 of the one-way analysis of variance (ANOVA) with SOS scores as the response variable, and the two realms as the predictor. Then we showed how the R^2 of different clades varied with their stem ages to evaluate the temporal changes in the contribution of different clades on realm divisions. To explore the major taxa driving realm divisions, we extracted the clades with the highest R^2 (90 quantile) for each pair of realms. The node-based analysis was conducted in R 3.6.1 [26] using the "nodiv" package (version 1.4.0) [41].

SUPPLEMENTARY RESULTS AND DISCUSSION

Comparison with floristic regionalization based on taxonomic beta diversity

Floristic realms and their relatedness based on taxonomic beta diversity are consistent with phylogenetic-based floristic realms with three main exceptions (Supplementary Fig. 10). First, subtropical East Asia was grouped into the Indo-Malesian realm when taxonomic beta diversity was used, but was grouped into the Holarctic realm when phylogenetic beta diversity was used. Second, Mexico was grouped into the Chile-Patagonian realm when taxonomic beta diversity was used but was grouped into the Neotropical realm when phylogenetic beta diversity was used. Third, (Australian, Chile-Patagonian) realms are grouped with (African, Indo-Malesian) realms, but were grouped with ((African, Indo-Malesian), (Neotropical, Chile-Patagonian)) realms when phylogenetic beta diversity was used.

Compared with the regionalization based on phylogenetic beta diversity, the regionalization based on taxonomic beta diversity may be more strongly influenced by contemporary climate and geographical isolation. For example, East Asia has similar climate with the Indo-Malesian realm, while its flora is more similar to that of the Holarctic realm[50-52]. Similarly, Mexico shares a similar dry climate with the Chile-Patagonian realm[53], while its flora is more similar to the North American flora[54]. These inconsistencies in climatic and floristic similarities may explain why taxonomic and phylogenetic beta diversity identify them as different realms. We further find that contemporary climate has a higher explanatory power on the taxonomic-based division than on the phylogenetic-based division of these realms (Supplementary Fig. 16). The relationship among (Australian, Chile-Patagonian), (African, Indo-Malesian) and (Neotropical, Chile-Patagonian) realms may mainly reflect current geographical isolation when taxonomic beta diversity is used, while their relationship may reflect plate tectonics when phylogenetic beta diversity is used (see Fig. 2 for the historical process of plate tectonics). These results demonstrate that floristic regionalization based on phylogenetic beta diversity is less affected by contemporary climate and better captures the floristic evolutionary history compared to the taxonomic-based regionalization[50].

Comparison with the species-level floristic regionalization

A recent species-level floristic regionalization based on data of a fraction (ca. 20%)

of vascular plants divides the world into three realms, i.e. the Holarctic, Holotropical and Austral realms [32]. The Holarctic realm in [32] is similar to the Laurasian super-realm in our study, although the boundaries differ to some extent (Fig. 1). The Holotropical realm in [32] covers the African, Indo-Malesian and Neotropical realms and north Chile-Patagonian realm in our study. The Austral realm in [32] covers the Australian and Novozealandic realms and southern Chile-Patagonian realm. The different floristic groupings between these two studies may be due to: 1) we included more complete taxa than Carta et al. (2022); 2) we used angiosperms at genus level while Carta et al. used vascular plants at species level, including some fern and gymnosperm species in addition to angiosperms. Thus, we further 1) extracted the angiosperm genera included in Carta et al. (2022) (9905 angiosperm genera) and 2) included both angiosperm and gymnosperm genera. Then we conducted the genus-level regionalization as the methods reported in our study. We found that both incomplete sampling and including gymnosperms did not change our floristic maps (Figure S8). These results suggests that the differences between our results and Carta et al. (2022) may reflect the difference in the taxonomic levels used for regionalization.

Notably, we identified eight realms using genus-level phylogenetic similarity, in contrasting with species-level regionalization by Carta et al. (2022) which identified 3 realms. In the previous regionalization schemes such as Engler's [55] or Takhtajan's [29], the global-scale floristic classification is usually based upon endemic families and genera rather than endemic species [29]. Although the methods for defining floristic clusters are different between our work and the previous regionalization schemes based on taxa endemism, we kept the same taxonomic level when conducting floristic maps. As a result, the number of floristic realms identified by the genus-level regionalization is similar to that in the previous regionalization schemes [29,56] and the boundaries well reflect the spatial divergences and the underlying evolutionary history in world's flora assemblages [54]. The differences in regionalization using different taxonomic levels may indicate that genus assemblages of plants may better capture the phylogenetic turnover across space at large scale [29].

Robustness of regionalization based on UPGMA clustering

Recent studies suggest that regions along the edges of the identified floristic realms may contain mixed floristic components and might form a "transitional zone"

[24,29]. Interestingly, our silhouette analysis for results based on the UPGMA methods shows that most GSUs near the edges of floristic realms are assigned to a realm with low uncertainty (Supplementary Fig. 2). We further identified the potential “transitional zone” which may be ignored in the UPGMA clustering by using fuzzy c-means as an alternative clustering method. We found that the floristic realms generated by the fuzzy c-means method are generally consistent with those generated by the UPGMA clustering, further indicating that the floristic realms are identified with low uncertainty (Supplementary Fig. 17). Even though, two differences were identified between the results based on the UPGMA and fuzzy c-means methods: 1) North America is clustered as a realm by the fuzzy c-means method but as a sub-realm in the Holarctic realm in UPGMA analysis; 2) Chile-Patagonian realm is not distinguished from Neotropical realm in the fuzzy c-means analysis. The fuzzy c-means method generates clusters based on differences among GSUs, while the UPGMA method generates clusters based on the differences between GSU groups and ignores the variations within groups [34,35]. The reasons for the difference between results of these two methods are likely because of a high proportion of Neotropical genera (Supplementary Data 5, also see Supplementary Fig. 19) in these regions (i.e. North America and Chile-Patagonia). These results indicate that the identification of these realms/sub-realms may have lower confidence compared with the other realms, suggesting that further investigations on the boundaries of floristic regions in the New World are needed in future studies.

Because compared with the UPGMA method, the fuzzy c-means method requires the user to specify a number of initial clusters (k) and the degree of fuzziness when assigning the membership of each GSU[35]. The setup of k and fuzziness is generally ambiguous and lack evaluations on the robustness of the choices. Furthermore, the fuzzy c-means do not generate a dendrogram showing the relationships between identified floristic regions as the UPGMA method does, and hence cannot be used to define floristic regions within each identified realm. This limits the ability of the fuzzy c-means method to evaluate the evolutionary history of the divisions between floristic realms. We therefore included the results based on the UPGMA clustering in the main text and those based the fuzzy c-means clustering method in the supplementary materials for comparison.

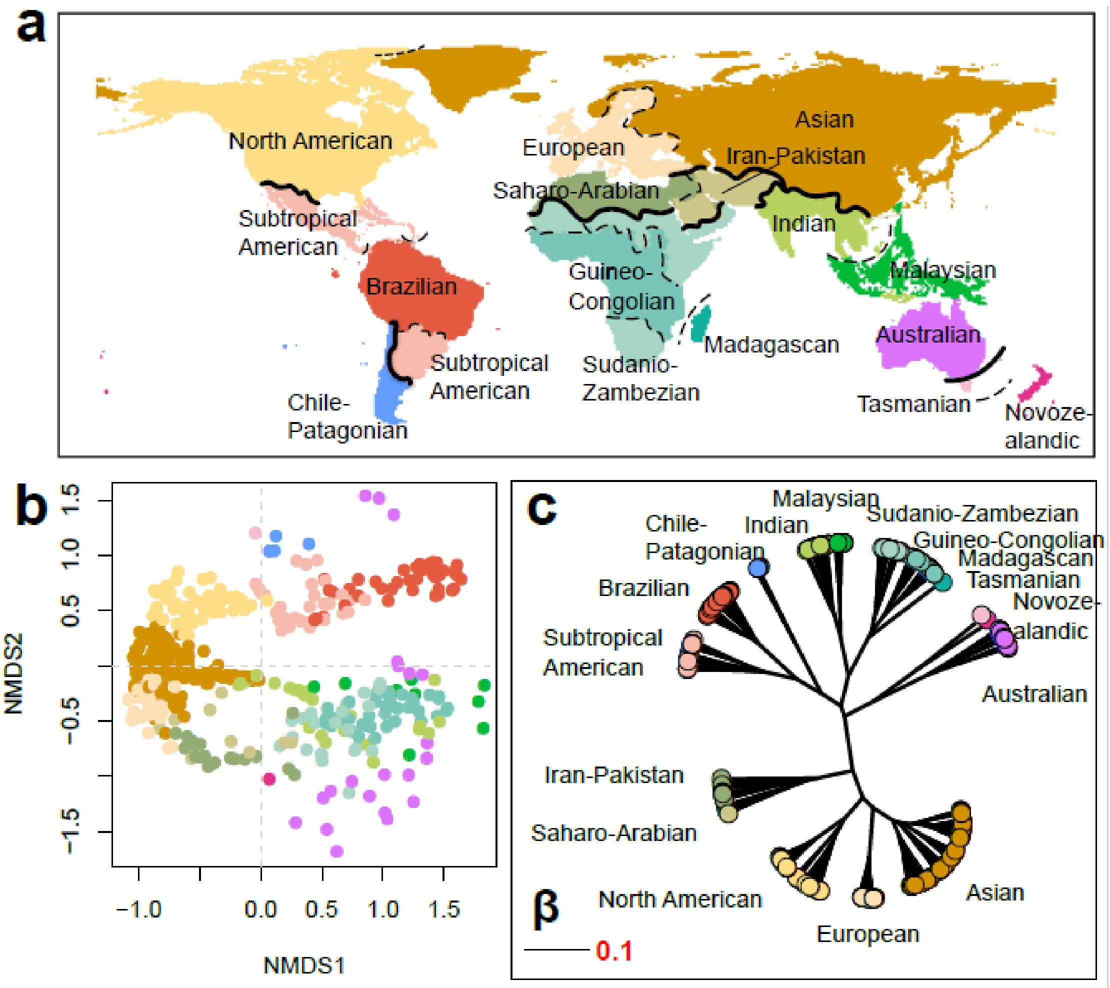

Supplementary Fig. 1 The boundaries and relationships between the 16 floristic sub-realms of the world. (a) Boundaries of the eight floristic realms (shown in solid line) and 16 sub-realms (shown in different colors), respectively. These boundaries were also shown in Figure 1. **(b)** The scatter plot shows the dissimilarities in the phylogenetic compositions between different geographic standard units (GSU) generated using non-metric multidimensional scaling (NMDS) ordination. **(c)** The unrooted dendrogram depicts the relationships among floristic sub-realms evaluated using UPGMA clustering method based on phylogenetic beta diversity between sub-realms. The scale bar in the dendrogram shows the dissimilarity between realms. Each tip in the dendrogram and each point in the scatter plot represents a geographic standard unit and the colors indicate the floristic realms that they belong to. Source data are provided as a Source Data file.

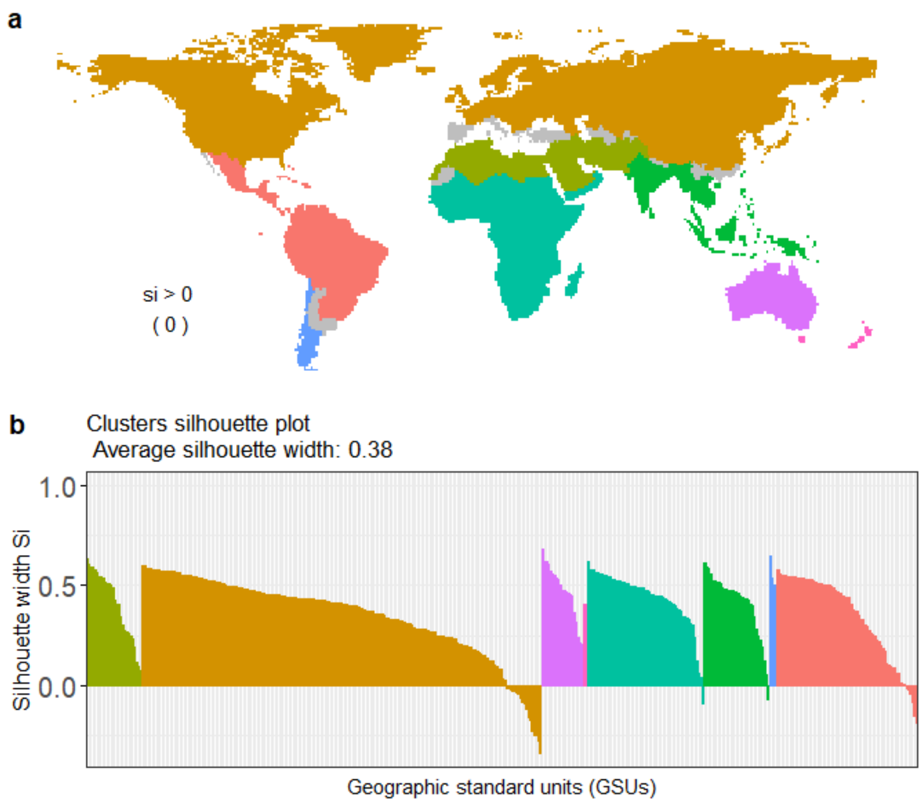

Supplementary Fig. 2 The performance of UPGMA clustering of phylogenetic beta diversity values evaluated using the silhouette width. Geographic units (GSU) where silhouette widths are lower than a certain threshold cannot be assigned to a single realm with high confidence, which are considered as uncertain regions. **a)** The identified uncertain regions (shown in gray) where silhouette widths are negative. **b)** The silhouette width for each GSU. Colors correspond to the floristic regions from this study as illustrated in **Fig. 1**. Source data are provided as a Source Data file.

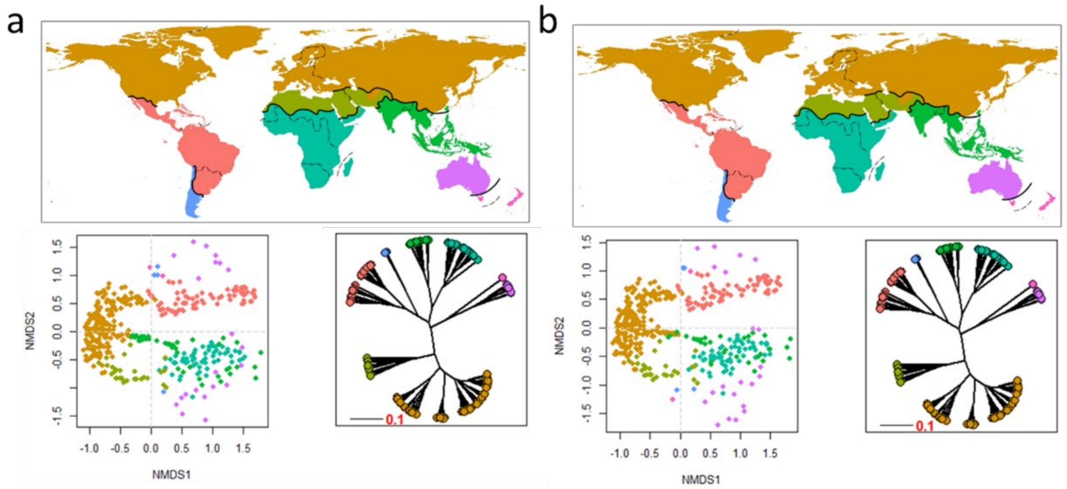

Supplementary Fig. 3 The floristic realms of the world and their relationships. The maps and dendrograms are generated based on the angiosperm phylogenies with (**a**) 140-150 Ma; (**b**) 140-259 Ma constraints for the angiosperms crown age. Boundaries of the eight floristic realms and 16 sub-realms show in solid and dashed lines, respectively. The scatter plots show the dissimilarities in the phylogenetic compositions between different geographic standard units generated using non-metric multidimensional scaling (NMDS) ordination. The unrooted dendrogram depicts the relationships among floristic realms evaluated using UPGMA clustering method based on phylogenetic beta diversity between realms. The scale bar in the dendrogram shows the dissimilarity between realms. Each tip in the dendrogram and each point in the scatter plot represents a geographic standard unit and the colors indicate the floristic realms that they belong to. Source data are provided as a Source Data file.

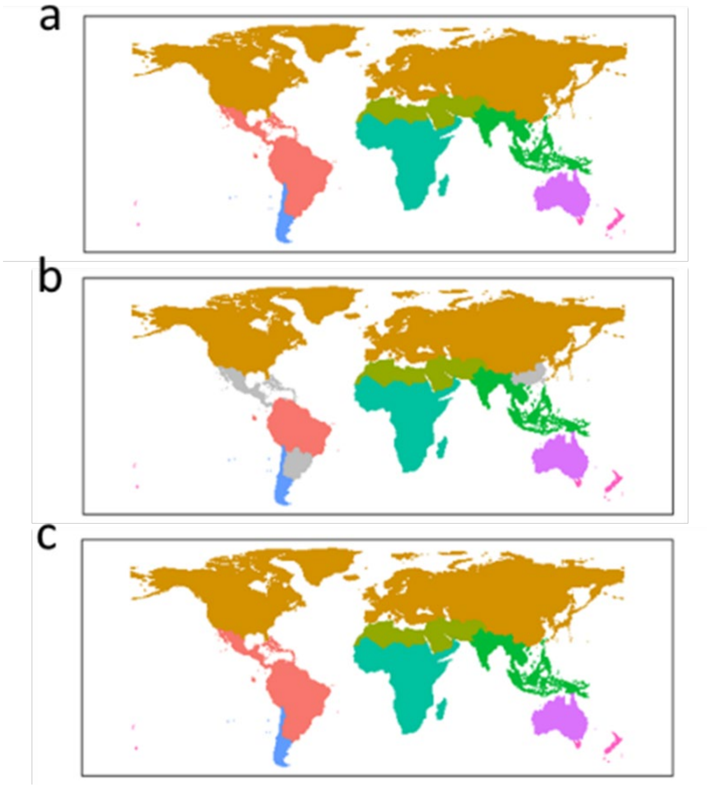

Supplementary Fig. 4 Comparison between the floristic maps based on the single maximum credibility tree. Phylogenetic trees are generated with different constraints for the angiosperms crown age. (**a**) 140-210 Ma; (**b**) 140-150 Ma and (**c**) 140-259 Ma. Regions differing between the results of the single maximum credibility tree and the individual trees are marked as gray.

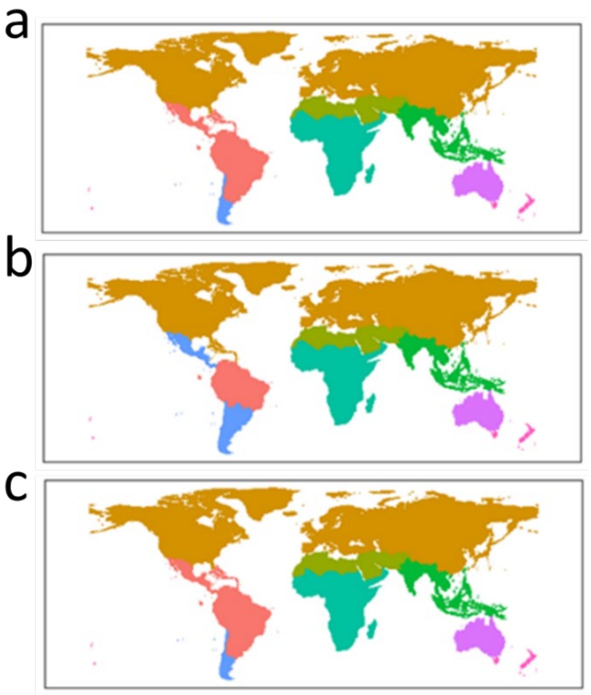

Supplementary Fig. 5 Floristic maps based on the molecular trees only including genera with molecular data. Phylogenetic trees are generated with different constraints for the angiosperms crown age. (**a**) 140-210 Ma; (**b**) 140-150 Ma and (**c**) 140-259 Ma.

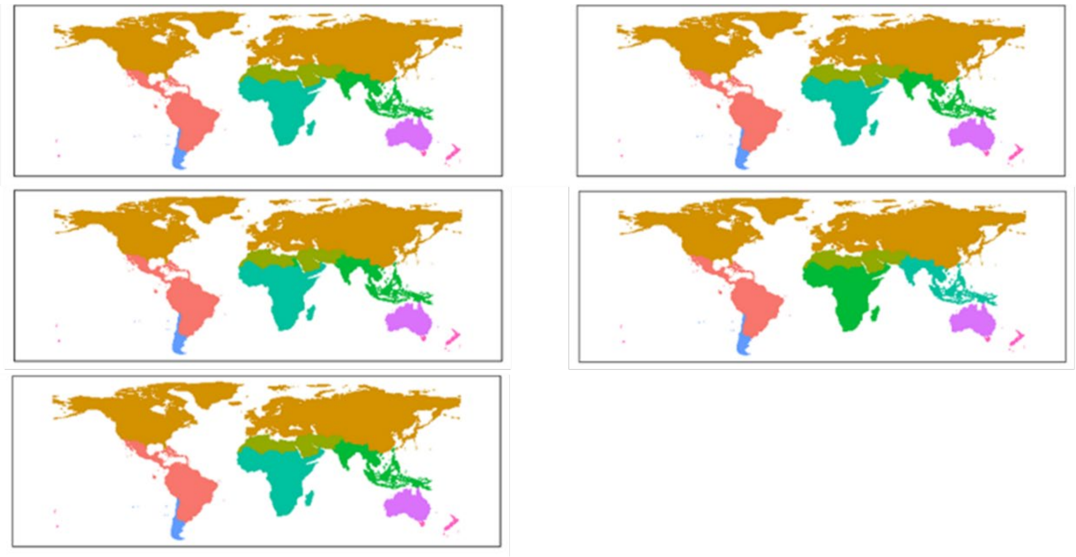

Supplementary Fig. 6 Floristic maps based on five randomly sampled individual trees drawn from the posterior distribution. Phylogenetic trees are generated with a constraint of 140-210 Ma for the angiosperm crown age.

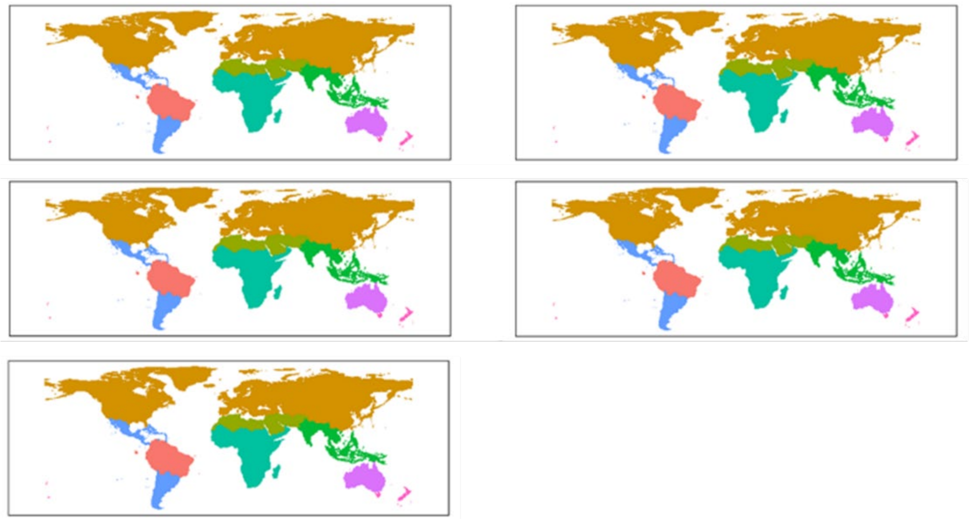

Supplementary Fig. 7 Floristic maps based on five randomly sampled individual trees drawn from the posterior distribution. Phylogenetic trees are generated with a constraint of 140-150 Ma for the angiosperm crown age.

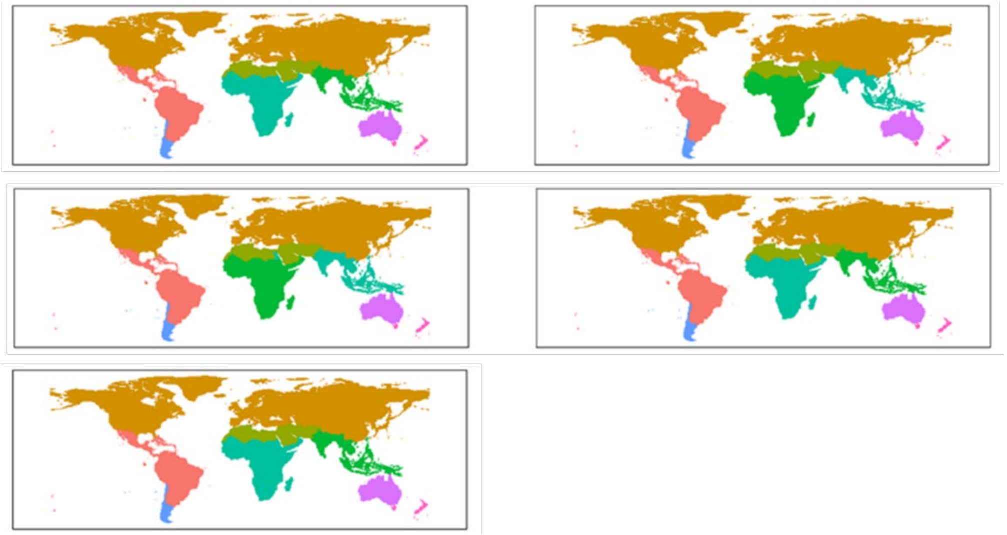

Supplementary Fig. 8 Floristic maps based on five randomly sampled individual trees drawn from the posterior distribution. Phylogenetic trees are generated with a constraint of 140-259 Ma for the angiosperm crown age.

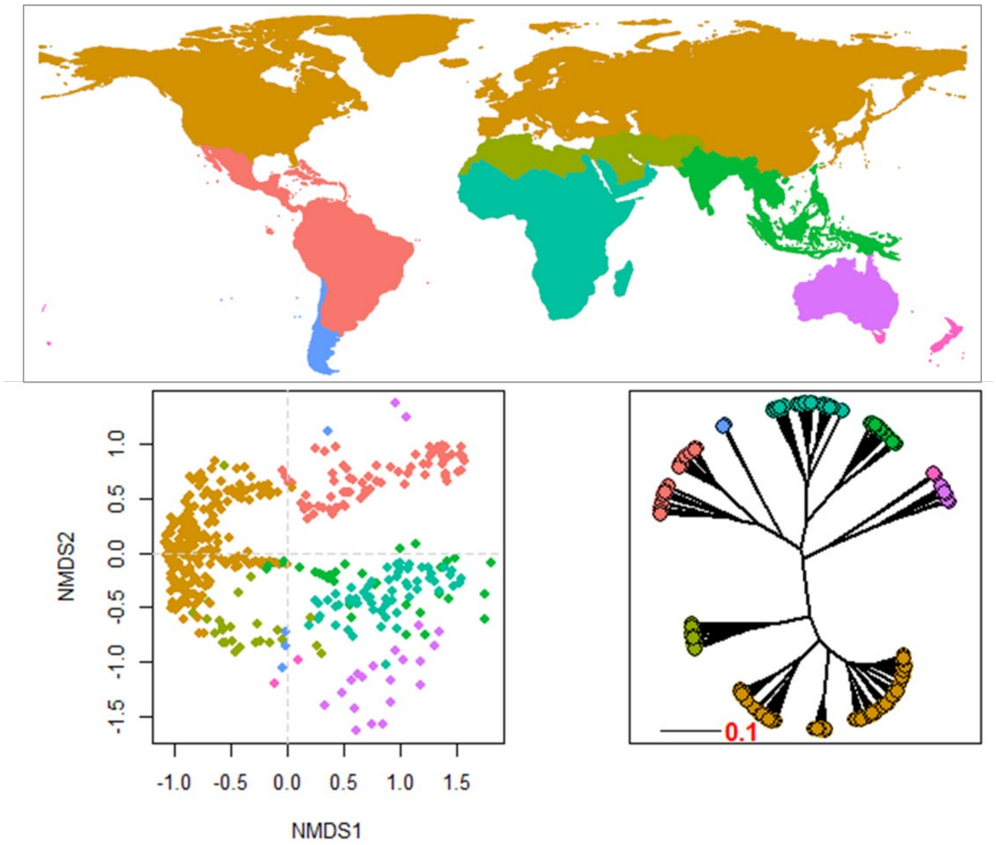

Supplementary Fig. 9 Biogeographic realms based on the angiosperm genera included in Carta et al. (2022). The unrooted dendrogram depicts the relationships among floristic realms evaluated using UPGMA clustering method based on phylogenetic beta diversity between realms. The scale bar in the dendrogram shows the dissimilarity between realms. The scatter plot shows the dissimilarities in the phylogenetic compositions between different geographic units generated using non-metric multidimensional scaling (NMDS) ordination. Each tip in the dendrogram and each point in the scatter plot represents a geographic standard unit and the colors indicate the floristic realms that they belong to. The realm boundaries are highly consistent with those reported in the main text. Source data are provided as a Source Data file.

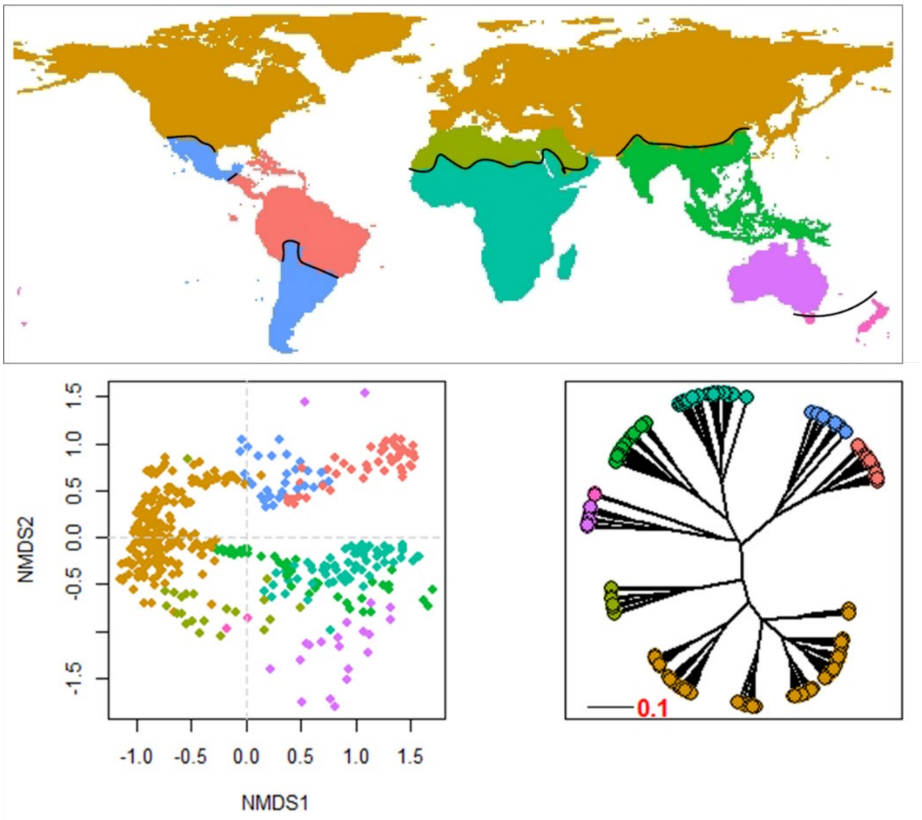

Supplementary Fig. 10 The map and dendrograms of realms based on taxonomic beta diversity. The unrooted dendrogram depicts the relationships among floristic realms evaluated using UPGMA clustering method based on phylogenetic beta diversity between realms. The scale bar in the dendrogram shows the dissimilarity between realms. The scatter plot shows the dissimilarities in the phylogenetic compositions between different geographic units generated using non-metric multidimensional scaling (NMDS) ordination. Each tip in the dendrogram and each point in the scatter plot represents a geographic standard unit and the colors indicate the floristic realms that they belong to. Source data are provided as a Source Data file.

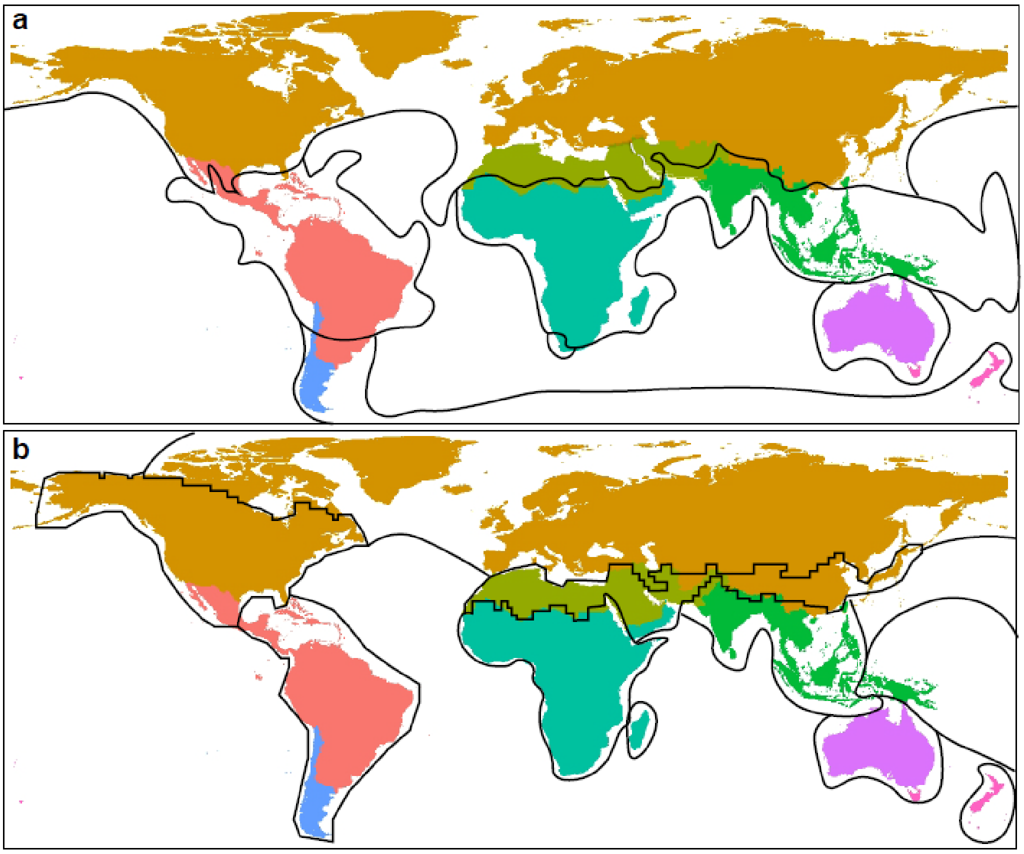

Supplementary Fig. 11 Comparison our floristic realms and sub-realms with a) Takhtajan's floristic map (Takhtajan 1986) and **b)** World's zoogeographic map based on phylo-distributional data for species of amphibians, birds and terrestrial mammals using UPGMA clustering (Holt *et al.* 2013). Colours correspond to the floristic realms from this study as illustrated in **Fig. 1**, and black lines delineate **a)** Takhtajan's floristic kingdoms or **b)** Holt *et al.*'s zoological realms.

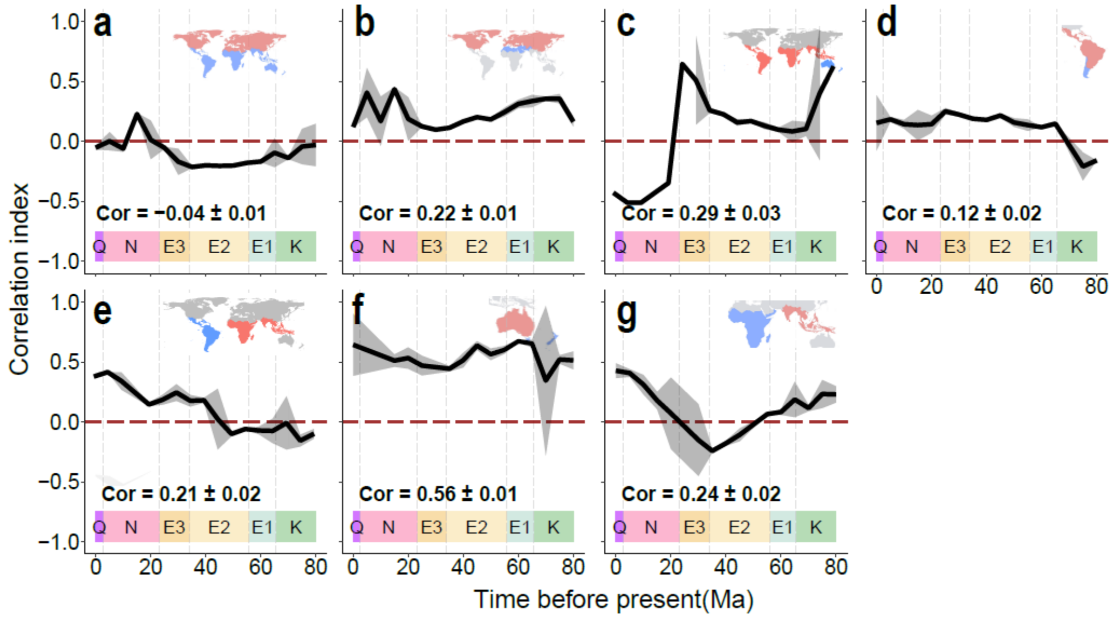

Supplementary Fig. 12 Pearson correlation indexes of geographic isolation and climate differences for each pair of floristic realms. The black lines and gray shaded areas represent the mean ± SE correlation values summarized every 5 Ma**. (a)** Gondwanan super-realm vs. Laurasian super-realm; **(b)** Holarctic realm vs. Saharo-Arabian realm; **(c)** (Australian, Novozealandic) realms vs. ((African, Indo-Malesian), (Neotropical, Chile-Patagonian)) realms; **(d)** Neotropical realm vs. Chile-Patagonian realm; **(e)** (African, Indo-Malesian) realms vs. (Neotropical, Chile-Patagonian) realms; **(f)** Australian realm vs. Novozealandic realm and **(g)** African realm vs. Indo-Malesian realm. Source data are provided as a Source Data file.

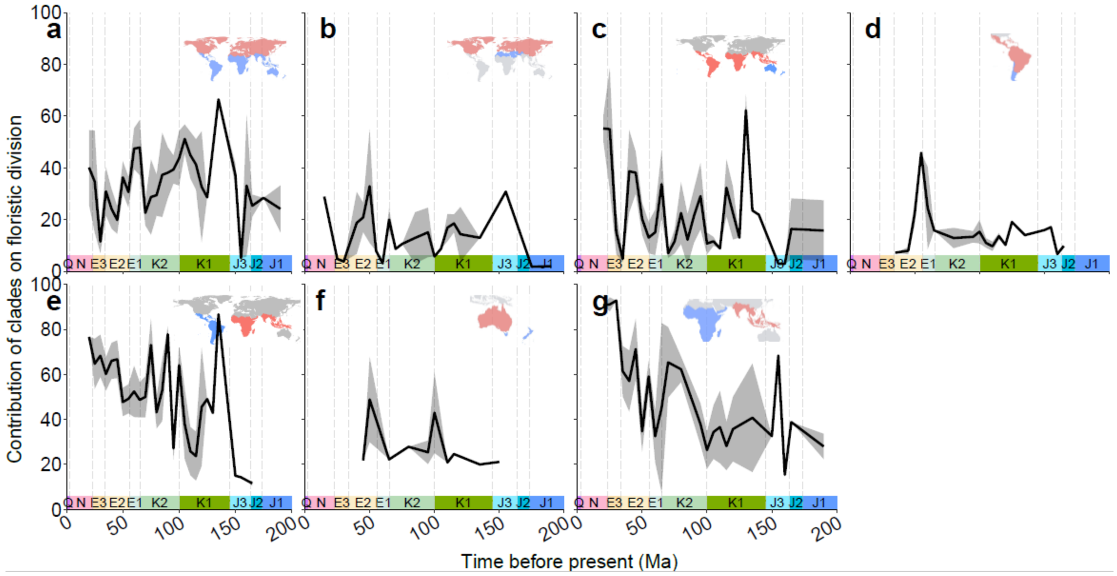

Supplementary Fig. 13 Contribution of different clades on the division between floristic realms (or cluster of realms). The division between realms (or cluster of realms) is shaped by clades whose descendant lineages show little overlap in their geographic distributions. Contribution of a clade is evaluated as the R^2 of the ANOVA with the SOS values as the response variable and the realms (or cluster of realms) as the explanatory variable (see **Methods** for more details). x axis represents the crown ages of the clades. The black lines and gray shaded areas represent the mean ± SE contribution of all clades summarized every 5 Ma**.** **(a)** Gondwanan super-realm vs. Laurasian super-realm; **(b)** Holarctic realm vs. Saharo-Arabian realm; **(c)** (Australian, Novozealandic) realms vs. ((African, Indo-Malesian), (Neotropical, Chile-Patagonian)) realms; **(d)** Neotropical realm vs. Chile-Patagonian realm; **(e)** (African, Indo-Malesian) realms vs. (Neotropical, Chile-Patagonian) realms; **(f)** Australian realm vs. Novozealandic realm and **(g)** African realm vs. Indo-Malesian realm. Source data are provided as a Source Data file.

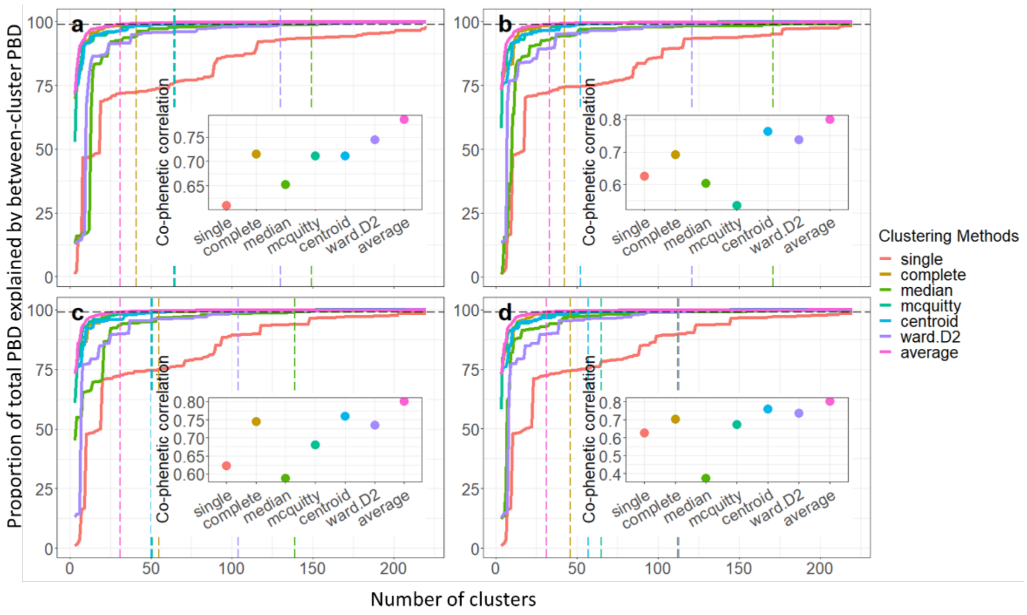

Supplementary Fig. 14 Comparison between the performances of different clustering methods. The distance matrix is represented by **a,** taxonomic beta diversity and **b-d,** phylogenetic beta diversity calculated using the angiosperm phylogenies with constraints of angiosperms crown age of **b,** 140-150 Ma, **c,** 140-210 Ma, **d,** 140-259 Ma, respectively. The main plots show the proportion (%) of the sum of beta diversity between all geographic standard units explained by the identified clusters (P_{beta}) using different clustering methods. The dash line represents the minimum number of clusters when the P_{beta} reached 99%. The subset on the bottom-right of each figure shows the differences in the co-phenetic coefficients of the seven clustering methods. The co-phenetic coefficients reflect the accuracy of different clustering methods (i.e. how well the clusters based on each clustering method correspond to the beta diversity between different geographic standard units). The colors of the dash lines, solid lines in the main figure and the points in the subset indicate different clustering methods. Source data are provided as a Source Data file.

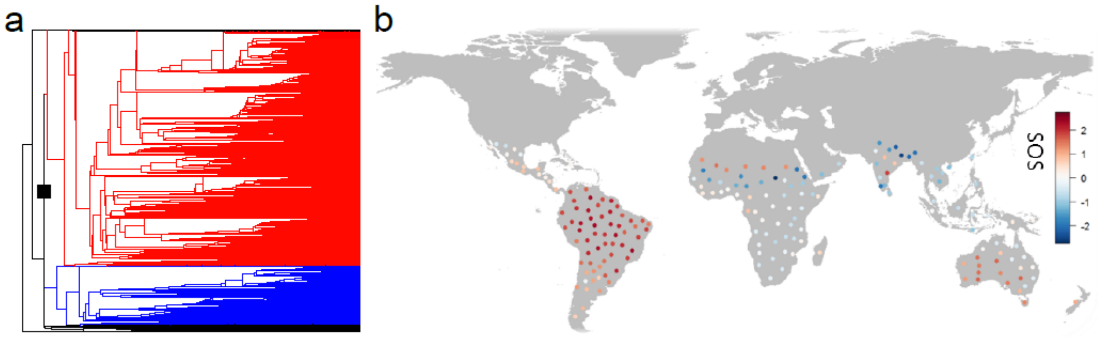

Supplementary Fig. 15 An example of node-based analysis. a, A given node (marked with the black square dot) with GND score > 0.65 (GND = 0.66 for this example), indicating significant distributional divergence between the two descendent lineages (shown in red and blue, respectively) of the node. The descendants (plant genera in our study) of the node are distributed in tropical regions. One descendent lineage occupies the Neotropical and the Australian region and the other distributes in India and along the southern limit of Sahara. The two descendent lineages overlap in tropical Africa and on the islands in Southeast Asia. Thus, this node has a major contribution for the division between the Neotropical and the other floristic realms. **b,** Patterns of specific overrepresentation score (SOS) for all the geographic standard units occupied by the descendent lineages. SOS values close to zero (shown in white) suggest that species from both descendent lineages are equally represented in each geographic unit; positive or negative SOS values (shown in red and blue, respectively, which is corresponding to the color of lineages in the phylogeny) indicate predominance of one of the two descendant lineages.

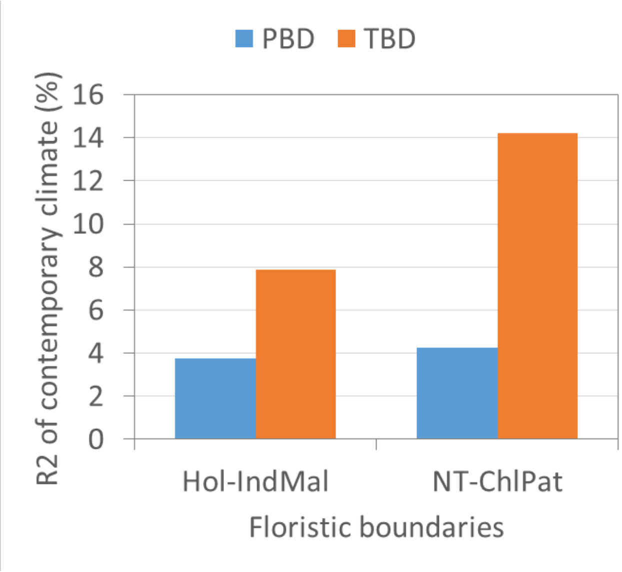

Supplementary Fig. 16 Effects of contemporary climate on the division of floristic realms identified using phylogenetic beta diversity (PBD) and taxonomic beta diversity (TBD). Bars show the R^2 (%) of contemporary climate in explaining phylogenetic beta diversity (blue) or in taxonomic beta diversity (orange) evaluated using Ordinary Least Square (OLS) models. For the division between the Holarctic and Indo-Malesian realms (Hol-IndMal), R^2 = 7.88% for TBD and R^2 = 3.74% for PBD; for the division between the Neotropical and Chile-Patagonian realms (NT-ChlPat), R^2 = 14.22% for TBD and R^2 = 4.26% for PBD. Source data are provided as a Source Data file.

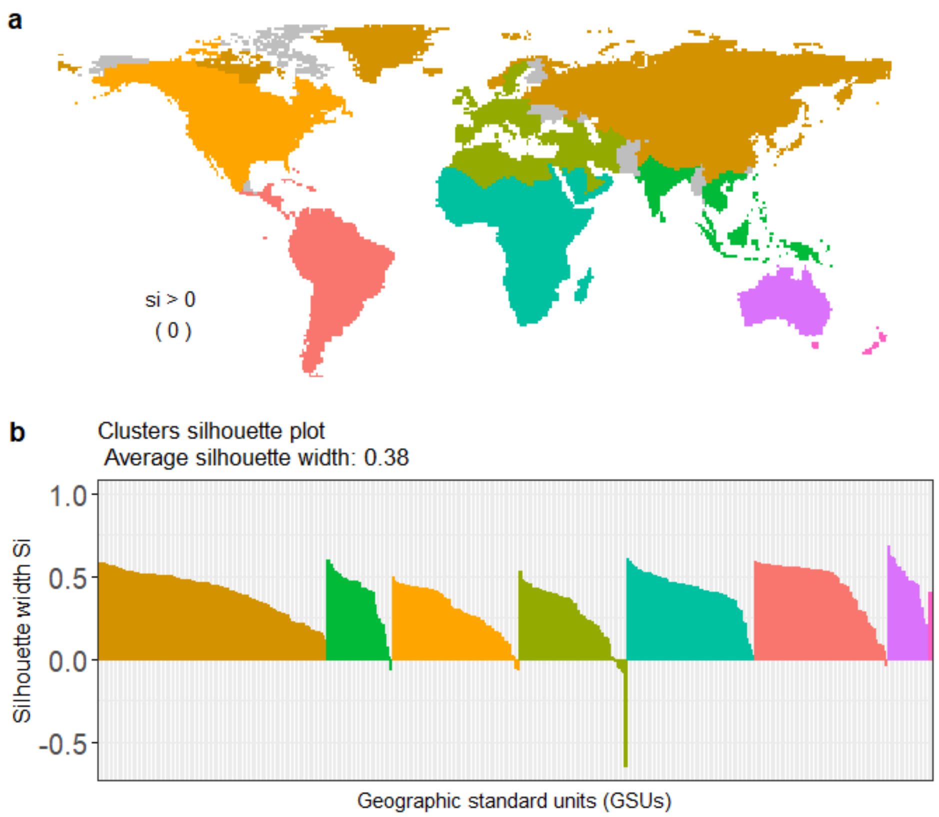

Supplementary Fig. 17 The performance of fuzzy c-means clustering of phylogenetic beta diversity values evaluated using the silhouette width. Geographic units (GSU) where silhouette widths are lower than a certain threshold cannot be assigned to a single realm with high confidence, which are considered as uncertain regions. **a)** The identified uncertain regions (shown in gray) where silhouette widths are negative. **b)** The silhouette width for each GSU. Colors correspond to the floristic regions from this study as illustrated in **Fig. 1**. Source data are provided as a Source Data file.

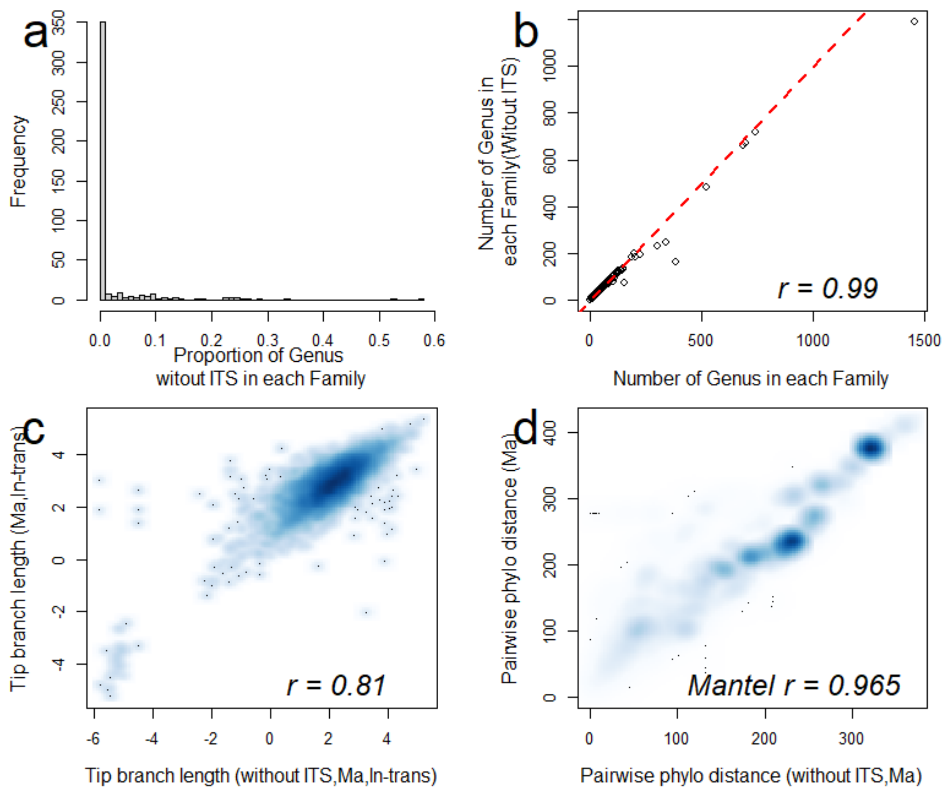

Supplementary Fig. 18 Comparison of phylogeny with/without ITS. a) histograms showing proportion of genera without ITS in each family. b) the number of genera in each family in the phylogeny with/without ITS. The red dash line indicates values where x axis and y axis are equal. c) the branch length of each genus in the dated phylogenies with/without ITS. Values are ln transformed. d) phylogenetic distance for pairwise genera estimated using branch length in the dated phylogenies with/without ITS. Source data are provided as a Source Data file.

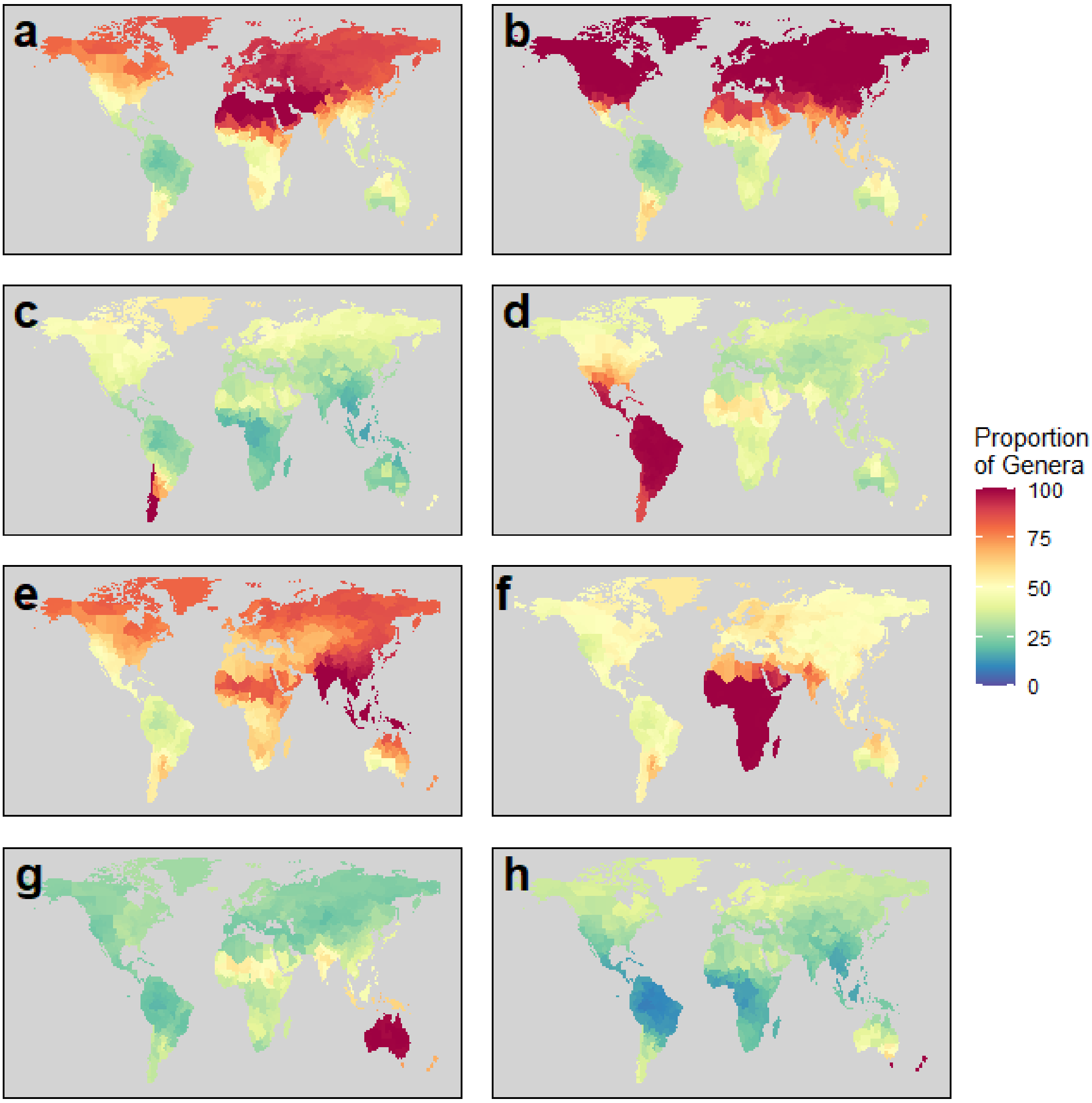

Supplementary Fig. 19 Natural ranges of 12,664 angiosperm genera. Color gradients indicate the proportions of genera in a geographic unit that are found in a certain floristic realm among all genera of that geographic unit. **a)** Saharo-Arabian realm, **b)** Holarctic realm, **c)** Chile-Patagonian realm, **d)** Neotropical realm, **e)** Indo-Malesian realm, **f)** African realm, **g)** Australian realm, **h)** Novozealandic realm. See **Supplementary Data 5** for detailed numbers of endemic genera within each floristic realm and the numbers of shared genera among different realms.

Supplementary Table 1 World floristic realms and sub-realms found here and in previous studies. Information for realms is shown in bold font.

ID	Name of floristic regions	Geographic ranges	Floristic regions proposed by Takhtajian (1986)	Zoological regions proposed by Holt *et al.* (2013)
**I**	**Saharo-Arabian Realm**	**North Africa, The Arabian Peninsula, Iran, and Pakistan**	**Saharo-Arabian Region，Iran part in Iran-Turanian Region, Pakistan part in Sudanio-Zambezian Region**	**Saharo-Arabian realm**
1	Iran-Pakistan Sub-realm	Iran and Pakistan	Iran part in Iran-Turanian Region, Pakistan part in Sudanio-Zambezian Region	Western Saharo-Arabian realm
2	Saharo-Arabian Sub-realm	Western Sahara, Morocco, Algeria, Tunisia, Egypt, Libya, and Saudi Arabia	Saharo-Arabian Region	Eastern Saharo-Arabian realm
**II**	**Holarctic Realm**	**Central Asia, Russia, East Asia, Europe, and North America**	**Holarctic Kingdom (exclude Saharo-Arabian Region)**	**Palearctic, Nearctic and Sino-Japanese realms**
3	North American Sub-realm	The United States and Canada	North American Atlantic Region, Rocky Mountain Region, Madrean Region and the Arctic Archipelago in Circumboreal Region	Nearctic realms
4	European Sub-realm	Europe and Turkey	Mediterranean Region，The European part of Circumboreal Region and Turkey in Iran-Turanian Region	West of the Palearctic realm
5	Asian Sub-realm	Central Asia, Russia, East Asia, Norway, and Greenland	North Asia, Russia, Norway and Greenland in Circumboreal Region, Eastern Asiatic Region and most parts of Iran-	East of the Palearctic realm and Sino-Japanese realm

			Turanian Region	
**III**	**Chile-Patagonian Realm**	**Chile and southern Argentina**	**Chile-Patagonian Region**	**South of the Neotropical realm**
6	Chile-Patagonian Sub-realm	Chile and southern Argentina	Chile-Patagonian Region	Southern South American region
**IV**	**Neotropical Realm**	**Mexico, Central America, Northern and Central South America**	**Neotropical Kingdom**	**Northern South American region, Amazonian, Panamanian and Mexican regions**
7	Brazilian Sub-realm	Central and northern Brazil, Bolivia, Peru, Ecuador, Colombia, Venezuela, Guyana, Suriname, and French Guiana	Amazonian Region，Guayana Highlands Region，North Brazilian Region and Andean Region	Northern South American region and Amazonian region
8	Subtropical American Sub-realm	South Brazil, north Paraguay, Uruguay and Argentina, Mexico, the Caribbean Islands, and Central America	Caribbean Region and South Brazilian Region	Panamanian and Mexican regions
**V**	**Indo-Malesian Realm**	**India, Sri Lanka, Bangladesh, Indo-China Peninsula, Malaysia, and New Guinea**	**Indo-Malesian Sub-kingdom**	**Oriental and Oceanian realms**
9	Malaysian Sub-realm	Philippines, Malaysia, and the islands of New Guinea	Malesian Region	Eastern oriental region and Indo-malayan ad Papua-Melanesian regions
10	Indian Sub-realm	India and Sri Lanka, Indo-China Peninsula, Hainan Island	Indochinese Region and Indian Region	Western oriental region
**VI**	**African Realm**	**Sub-Saharan Africa and southern Arabian Peninsula**	**African Sub-kingdom，Madagascan Sub-kingdom, and Cape Kingdom**	**Afrotropical and Madagascan realms**

11	Madagascan Sub-realm	Madagascar	Madagascan Region	Madagascan realms
12	Guineo-Congolian Sub-realm	Upper Guinea Plateau, Lower Guinea Plateau, Congo Basin, Tanzania, and Mozambique	Guineo-Congolian Region, north Uzambara-Zululand Region, north Karroo-Namib Region and the tropical African parts of Sudanio-Zambezian Region	Guineo-Congolian Region
13	Sudanio-Zambezian Sub-realm	Sudan Grassland, Ethiopian Highland, Yemen and Oman in the southern Arabian Peninsula, South Africa, Namibia, Botswana, Swaziland, and Lesotho	Sudanio-Zambezian Region, south Uzambara-Zululand Region, south Karroo-Namib Region and Cape region	African region
**VII**	**Australian Realm**	**Continent of Australia**	**Australian Kingdom**	**Australian realm**
14	Australian Sub-realm	Continent of Australia	Northeast Australian Region, Southwest Australian Region, and Central Australian Region	Australian Region
**VIII**	**Novozealandic Realm**	**New Zealand and Tasmania**	**Neozeylandic Region and Tasmania in Northeast Australian Region**	**Novozelandic realm**
15	Novozealandic Sub-realm	New Zealand	Neozeylandic Region	Novozealandic Region
16	Tasmanian Sub-realm	Tasmania	Tasmania in Northeast Australian Region	Tasmania in Australian Region

SUPPLEMENTARY REFERENCES

1 Smith, S. A. & Brown, J. W. Constructing a broadly inclusive seed plant phylogeny. *American journal of botany* **105**, 302-314, doi:10.1002/ajb2.1019 (2018).

2 Zanne, A. E. *et al.* Three keys to the radiation of angiosperms into freezing environments. *Nature* **506**, 89-92, doi:10.1038/nature12872 (2014).

3 Silvestro, D. & Michalak, I. raxmlGUI: a graphical front-end for RAxML. *Organisms Diversity & Evolution* **12**, 335-337, doi:10.1007/s13127-011-0056-0 (2012).

4 Smith, S. A. & O'Meara, B. C. treePL: divergence time estimation using penalized likelihood for large phylogenies. *Bioinformatics* **28**, 2689-2690, doi:10.1093/bioinformatics/bts492 (2012).

5 Foster, C. S. P. *et al.* Evaluating the Impact of Genomic Data and Priors on Bayesian Estimates of the Angiosperm Evolutionary Timescale. *Systematic Biology* **66**, 338-351, doi:10.1093/sysbio/syw086 (2017).

6 Magallón, S. Using fossils to break long branches in molecular dating: a comparison of relaxed clocks applied to the origin of angiosperms. *Systematic Biology* **59**, 384-399 (2010).

7 Wu, Z. *et al.* A precise chloroplast genome of Nelumbo nucifera (Nelumbonaceae) evaluated with Sanger, Illumina MiSeq, and PacBio RS II sequencing platforms: insight into the plastid evolution of basal eudicots. *BMC Plant Biology* **14**, 1-14 (2014).

8 Barba-Montoya, J., dos Reis, M., Schneider, H., Donoghue, P. C. J. & Yang, Z. Constraining uncertainty in the timescale of angiosperm evolution and the veracity of a Cretaceous Terrestrial Revolution. *New Phytologist* **218**, 819-834, doi:https://doi.org/10.1111/nph.15011 (2018).

9 Stevens, P. *Angiosperm phylogeny website. Version 13*, <http://www.mobot.org/MOBOT/research/APweb/> (2015).

10 Silvestro, D., Cascales-Miñana, B., Bacon, C. D. & Antonelli, A. Revisiting the origin and diversification of vascular plants through a comprehensive Bayesian analysis of the fossil record. *New Phytologist* **207**, 425-436, doi:https://doi.org/10.1111/nph.13247 (2015).

11 Iltis, H. H., Hall, J. C., Cochrane, T. S. & Sytsma, K. J. Studies in the Cleomaceae I. On the Separate Recognition of Capparaceae, Cleomaceae, and Brassicaceae1. *Annals of the Missouri Botanical Garden* **98**, 28-36 (2011).

12 Kuhn, T. S., Mooers, A. Ø. & Thomas, G. H. A simple polytomy resolver for dated phylogenies. *Methods in Ecology & Evolution* **2**, 427-436, doi:https://doi.org/10.1111/j.2041-210X.2011.00103.x (2011).

13 Drummond, A. J., Suchard, M. A., Xie, D. & Rambaut, A. Bayesian Phylogenetics with BEAUti and the BEAST 1.7. *Molecular Biology and Evolution* **29**, 1969-1973, doi:10.1093/molbev/mss075 (2012).

14 Rambaut, A. & Drummond, A. J. Tracer v1.4. *Encyclopedia of Atmospheric Sciences* **141**, 2297–2305 (2007).

15 Jetz, W., Thomas, G. H., Joy, J. B., Hartmann, K. & Mooers, A. O. The global diversity of birds in space and time. *Nature* **491**, 444-448, doi:10.1038/nature11631 (2012).

16 Li, H.-T. *et al.* Origin of angiosperms and the puzzle of the Jurassic gap. *Nature plants* **5**, 461-470 (2019).

17 Ramírez-Barahona, S., Sauquet, H. & Magallón, S. The delayed and geographically heterogeneous diversification of flowering plant families. *Nature Ecology & Evolution* **4**, 1232-1238, doi:10.1038/s41559-020-1241-3 (2020).

18 Janssens, S. B. *et al.* A large-scale species level dated angiosperm phylogeny for evolutionary and ecological analyses. *Biodiversity data journal* **8**, e39677-e39677, doi:10.3897/BDJ.8.e39677 (2020).

19 Shrestha, N. *et al.* Global patterns of Rhododendron diversity: The role of evolutionary time and diversification rates. *Global Ecology and Biogeography* **27**, 913-924, doi:https://doi.org/10.1111/geb.12750 (2018).

20 Wang, Y. *et al.* Global distribution and evolutionary transitions of angiosperm sexual systems. *Ecology Letters* **24**, 1835-1847, doi:https://doi.org/10.1111/ele.13815 (2021).

21 Powo. *Plants of the World Online. Facilitated by the Royal Botanic Gardens, Kew.* , <http://www.plantsoftheworldonline.org/> (2023).

22 Simpson, G. G. Mammals and the nature of continents. *American Journal of Science* **241**, 1-31, doi:10.2475/ajs.241.1.1 (1943).

23 Lennon, J. J., Koleff, P., GreenwooD, J. J. D. & Gaston, K. J. The geographical structure of British bird distributions: diversity, spatial turnover and scale. *Journal of Animal Ecology* **70**, 966-979, doi:https://doi.org/10.1046/j.0021-8790.2001.00563.x (2001).

24 Kreft, H. & Jetz, W. A framework for delineating biogeographical regions based on species

distributions. *Journal of Biogeography* **37**, 2029-2053 (2010).

25 Holt, B. G. *et al.* An Update of Wallace's Zoogeographic Regions of the World. *Science* **339**, 74-78 (2013).

26 Team, R. C. (R Foundation for Statistical Computing, Vienna, Austria, 2021).

27 Sneath, P. H. A. & Sokal, R. R. *Numerical Taxonomy: The Principles and Practice of Numerical Classification*. 278 (Freeman, 1973).

28 Kruskal, J. B. Nonmetric multidimensional scaling: A numerical method. *Psychometrika* **29**, 115-129, doi:10.1007/BF02289694 (1964).

29 Takhtajan, A., Crovello, T. J. & Cronquist, A. *Floristic Regions of The World*. 544 (University of California press, 1986).

30 Morrone, J. J. Biogeographical regionalisation of the world: a reappraisal. *Aust. Syst. Bot.* **28**, 81 (2015).

31 Lomolino, M. V., Riddle, B. R. & Whittaker, R. J. *Biogeography*. (Sinauer Associates, 2016).

32 Carta, A., Peruzzi, L. & Ramírez-Barahona, S. A global phylogenetic regionalization of vascular plants reveals a deep split between Gondwanan and Laurasian biotas. *New Phytologist* **233**, 1494-1504, doi:https://doi.org/10.1111/nph.17844 (2022).

33 Rousseeuw, P. J. Silhouettes: a graphical aid to the interpretation and validation of cluster analysis. *Journal of computational and applied mathematics* **20**, 53-65 (1987).

34 Pal, N. R., Bezdek, J. C. & Hathaway, R. J. Sequential competitive learning and the fuzzy c-means clustering algorithms. *Neural Networks* **9**, 787-796 (1996).

35 Bezdek, J. C. *Pattern recognition with fuzzy objective function algorithms*. (Springer Science & Business Media, 2013).

36 Maechler, M., Rousseeuw, P., Struyf, A., Hubert, M. & Hornik, K. Cluster: cluster analysis basics and extensions. *R package version* **1**, 56 (2012).

37 Daru, B. H., Michelle, V. D. B., Davies, T. J. & Franklin, J. Unravelling the evolutionary origins of biogeographic assemblages. *Diversity and Distributions* **24**, 313-324 (2018).

38 He, J., Lin, S., Li, J., Yu, J. & Jiang, H. Evolutionary history of zoogeographical regions surrounding the Tibetan Plateau. *Communications Biology* **3**, 1-9 (2020).

39 Mazel, F. *et al.* Global patterns of β-diversity along the phylogenetic time-scale: The role of climate and plate tectonics. *Global Ecology and Biogeography* **26**, 1211-1221 (2017).

40 Groussin, M. *et al.* Unraveling the processes shaping mammalian gut microbiomes over evolutionary time. *Nature Communications* **8**, 1-12 (2017).

41 Borregaard, M. K. *et al.* Node-based analysis of species distributions. *Methods in Ecology & Evolution* **5**, 1225-1235 (2015).

42 Scotese, C. R. *PALEOMAP paleodigital elevation models (PaleoDEMS) for the Phanerozoic.* , <https://www. earthbyte. org/paleodem-resource-scotese-and-wright-2018 > (2018).

43 Hagen, O. *et al.* Mountain building, climate cooling and the richness of cold-adapted plants in the Northern Hemisphere. *Journal of Biogeography* **46**, 1792-1807, doi:https://doi.org/10.1111/jbi.13653 (2019).

44 Descombes, P., Leprieur, F., Albouy, C., Heine, C. & Pellissier, L. Spatial imprints of plate tectonics on extant richness of terrestrial vertebrates. *Journal of Biogeography* **44**, 1185-1197 (2017).

45 Boyden, J. A. *et al.* in *Geoinformatics: Cyberinfrastructure for the Solid Earth Sciences* (eds Chaitanya Baru & G. Randy Keller) 95-114 (Cambridge University Press, 2011).

46 Dijkstra, E. W. A note on two problems in connexion with graphs. *Numerische Mathematik* **1**, 269-271 (1959).

47 van Etten, J. R Package gdistance: Distances and Routes on Geographical Grids. *Journal of Statistical Software* **76**, 21, doi:10.18637/jss.v076.i13 (2017).

48 Scotese, C. R. *Some Thoughts on Global Climate Change: The Transition for Icehouse to Hothouse Conditions*. (PALEOMAP project, 2016).

49 Boucot, A. J., Xu, C. & Scotese, C. R. *Phanerozoic Paleoclimate: An Atlas of Lithologic Indicators of Climate*. (SEPM, 2013).

50 Ye, J. *et al.* Phylogenetic delineation of regional biota: A case study of the Chinese flora. *Molecular phylogenetics and evolution* **135**, 222-229, doi:10.1016/j.ympev.2019.03.011 (2019).

51 Wen, J. Evolution of eastern Asian and eastern North American disjunct distributions in flowering plants. *Annual Review of Ecology and Systematics* **30**, 421-455 (1999).

52 Xing, Y., Gandolfo, M. A. & Linder, H. P. The Cenozoic biogeographical evolution of woody angiosperms inferred from fossil distributions. *Global Ecology and Biogeography* **24**, 1290-1301 (2015).

53 Peel, M. C., Finlayson, B. L. & McMahon, T. A. Updated world map of the Köppen-Geiger

climate classification. *Hydrology and Earth System Sciences* **11**, 1633-1644, doi:10.5194/hess-11-1633-2007 (2007).
54 Cox, B. The biogeographic regions reconsidered. *Journal of Biogeography* **28**, 511-523 (2001).
55 Engler, A. & Prantl, K. A. E. *Die Natürlichen Pflanzenfamilien nebst ihren Gattungen und wichtigeren Arten: insbesondere den Nutzpflanzen*. (Wilhelm Engelmann, 1899).
56 Good, R. *Geography of the Flowering Plants*. (Longmans, 1964).
