# Supplementary material for: An updated floristic map of the world
Source: Nat Commun. 2023 May 30;14:2990. doi: 10.1038/s41467-023-38375-y (PMC10229591; doi:10.1038/s41467-023-38375-y)
Supplement: Supplementary file 3 — Description of Additional Supplementary Files [file 41467_2023_38375_MOESM3_ESM.pdf]

Description of Additional Supplementary Files

File Name: Supplementary Data 1

Description: Data sources for Global distributions of angiosperm

File Name: Supplementary Data 2

Description: Global distributions of angiosperm genera

File Name: Supplementary Data 3

Description: Clades with the highest contributions on floristic divergences

File Name: Supplementary Data 4

Description: The list of non-monophyletic families

File Name: Supplementary Data 5

Description: The number of genera that are endemic to each realm and the number of genera that are distributed across more than one realm

File Name: Supplementary Data 6

Description: Genus list & Accession Numbers
